# Large Language Models for Multidisciplinary Tumor Board Decision-Making in Primary Liver Tumors: A Retrospective Single-Center Study

**DOI:** 10.3390/cancers18132175

**Published:** 2026-07-07

**Authors:** Julian Palzer, Alexander Genchev, Esref Belger, Clara Antonia Weigle, Bengt Arne Wiemann, Philipp Tessmer, Mohamad Murad, Marie-Luise Berres, Franziska Alexandra Meister, Iakovos Amygdalos, Philipp Bruners, Daniel Truhn, Ahmed Allam Mohamed, Florian Wolfgang Rudolf Vondran, Felix Oldhafer, Oliver Beetz

**Affiliations:** 1 Department of General, Visceral, Pediatric and Transplant Surgery, University Hospital RWTH Aachen, 52074 Aachen, Germany; 2 Centrum for Integrated Oncology, University Hospital RWTH Aachen, 52074 Aachen, Germany; 3 Department of Gastroenterology, University Hospital RWTH Aachen, 52074 Aachen, Germany; 4 Department of Radiology, University Hospital RWTH Aachen, 52074 Aachen, Germany; 5 Department of Radiation Oncology, University Hospital RWTH Aachen, 52074 Aachen, Germany

**Keywords:** artificial intelligence, large language models, LLM, ChatGPT, Claude, multidisciplinary tumor board, cancer, primary liver malignancies, hepatocellular carcinoma, cholangiocellular carcinoma

## Abstract

**Introduction**: Multidisciplinary tumor boards (MTBs) are the standard for oncologic decision-making but require substantial time and personnel resources. Large language models (LLMs) may support MTBs by generating structured, guideline-based recommendations. We evaluated concordance between MTB decisions and LLM recommendations in newly diagnosed cholangiocellular adenocarcinoma (CCA) and hepatocellular carcinoma (HCC). **Method**: In this retrospective single-center study, MTB protocols from 2022–2023 were screened. After exclusions, 50 CCA and 50 HCC cases were analyzed. Institutional MTB recommendations were compared with outputs from ChatGPT and Claude using identical structured clinical summaries available at the time of MTB presentation. Agreement was assessed using Cohen’s kappa, and correlation using Spearman’s rank coefficient. **Results**: For CCA, exact concordance between MTB and ChatGPT was 80%, with substantial agreement (k = 0.688) and strong correlation (r = 0.725; both *p* < 0.001). Concordance with Claude was at 56%. For HCC, concordance between MTB and ChatGPT was 66%, with substantial agreement (k = 0.604) and moderate correlation (r = 0.484; both *p* < 0.001). Concordance with Claude was only 38%, with fair agreement (k = 0.314, *p* < 0.001) and weak correlation (r = 0.086, *p* = 0.551). **Conclusions**: LLM-derived recommendations varied markedly by model. ChatGPT demonstrated substantial concordance, whereas Claude showed limited agreement. MTBs appeared more individualized, while LLM outputs were more guideline-oriented. These findings highlight the potential role of LLMs as supportive tools for structured clinical reasoning and guideline adherence. Prospective multicenter studies should evaluate real-time LLM integration into MTB workflows, efficiency, decision quality, and patient outcomes safely.

## Simple Summary

Multidisciplinary tumor boards (MTBs) are essential for developing individualized treatment strategies for patients with cancer by integrating expertise from multiple medical specialties. Recent advances in artificial intelligence have raised interest in whether large language models (LLMs) could support this process by generating structured, evidence-based treatment recommendations. In this study, we compared recommendations generated by two widely used LLMs with real-world decisions made by an MTB for patients with primary liver cancers. One model demonstrated substantial agreement with expert recommendations, whereas the other adopted a more cautious approach and showed lower concordance. These findings suggest that the performance of LLMs varies considerably between models and that selected systems may have the potential to support clinical reasoning and complement multidisciplinary decision-making. However, their recommendations require careful expert review, and prospective studies are needed to evaluate their impact on clinical workflows, decision quality, and patient outcomes before routine clinical implementation can be considered.

## 1. Introduction

Multidisciplinary tumor boards (MTBs) are central to oncologic treatment planning. They bring together specialists from different disciplines to formulate individualized treatment recommendations. Given the clinical burden presented by malignant tumors, MTBs are most commonly held weekly and usually take up one to one-and-a-half hours. In this period of time, all the attending physicians are bound due to their expertise, essential for critical decision making for individual patients fighting oncologic disease. This eventuates in a severe workload share attributed to MTBs, with multiple MTBs per week and even per day, depending on the size of the hospital [1]. Although these decisions are critical for the patient’s outcome, the time consumed by conference preparation, conference and post-conference is missing in the direct patient care, hence, creating a disadvantageous time ratio between conferences and patient care [2]. Previous efforts have established the implementation of various tools or preparative strategies, increasing the efficiency of MTBs [3,4]. With the advent of artificial intelligence (AI), which is increasingly used to improve the efficiency of clinical and administrative workflows, the question has arisen whether AI, and particularly Large Language Models (LLMs), could be of use in oncologic decision-making within an MTB setting. Hereby, relieving physicians while simultaneously creating more time for clinical care. The utility of LLMs as part of an MTB has been addressed in recent studies for various cancer entities [5,6]. Among such, retro- as well as prospective comparative analyses present concordance rates ranging from moderate [7] to fairly high rates of up to 76.4% [8]. In this study, we performed a retrospective single-center comparison of decisions made by an MTB and LLM-based recommendations derived from two different LLMs: ChatGPT and Claude. Hereby, addressing the utility and applicability of LLMs for decision-making on behalf of oncologic treatment decisions for patients diagnosed with the most common malignant primary liver tumors, cholangiocellular adenocarcinoma (CCA) and hepatocellular carcinoma (HCC), on the one hand, while comparing two LLMs of different nature for their feasibility regarding clinical decision making.

Depending on its anatomical localization, CCAs are distinguished in (1) intrahepatic CCA (iCCA), perihilar CCA or Klatskin tumors (pCCA), distal CCA (dCCA) and CCA localized in the gallbladder [9]. All of these subtypes were taken into account. Therapeutic options comprise primarily systemic immune and/or chemotherapy and surgical resection [10,11].

HCC represents the most common primary liver malignancy worldwide and typically develops in the context of chronic liver disease and cirrhosis. HCC management is inherently complex, as therapeutic decisions must balance tumor burden, hepatic functional reserve, and patient performance status. Accordingly, treatment allocation is guided by integrated staging systems, most prominently the Barcelona Clinic Liver Cancer (BCLC) classification, which links disease stage to evidence-based therapeutic recommendations [12]. Available treatment modalities range from curative approaches such as surgical resection, liver transplantation, and local ablation to transarterial therapies, stereotactic body radiotherapy (SBRT), and systemic treatment with targeted agents and immune checkpoint inhibitors [13,14]. Recent advances, particularly the introduction of immunotherapy-based combination regimens, have significantly improved outcomes in advanced disease and expanded the therapeutic landscape [15]. Additionally, emerging downstaging and conversion strategies aim to enable curative interventions in initially unresectable patients [16,17]. Together, this rapidly evolving and increasingly complex treatment paradigm places substantial demands on MTBs and underscores the potential value of LLM-assisted decision-support systems in optimizing clinical workflows and supporting evidence-based treatment decisions.

## 2. Method

### 2.1. Study Design

For this retrospective single-center analysis, a cohort of patients newly diagnosed with either CCA or HCC was identified from MTB protocols. The study followed ethical guidelines for research involving human subjects, with approval from the Ethics Committee of RWTH Aachen University under the number EK 26-088. The objective of the study was to evaluate agreement between LLM-derived recommendations and realword MTB decisions. Concordance was not interpreted as a measure of clinical correctness. Comparative analysis of LLM-generated and MTB-derived decisions was performed using real-world data in terms of the actual pseudonymized MTB registration forms. The LLM input included all pseudonymized clinical information documented in the registration forms, while the final therapeutic decision of the MTB was deliberately excluded to prevent potential bias. Importantly, only disease-related clinical information was provided to the LLMs, and no patient-identifying data (such as name, date of birth, or case identifiers) were included at any stage of the analysis. Specifically, the information provided to the LLMs was limited to patient age, ECOG-PS, and disease-specific clinical data, including tumor characteristics, staging, relevant diagnostic findings, as well as laboratory parameters, including liver function tests and variables relevant for the assessment of resectability.

At first, the LLMs were prompted to simulate an oncologic MTB decision for patients with cancers within the following frame work: (1) a direct treatment recommendation with a defined oncologic strategy, (2) conditional “if–then” recommendations linked to specific diagnostic findings if the results are still pending, (3) a request for additional diagnostic procedures only if essential for establishing a treatment decision, or (4) a combination of diagnostic clarification and definitive or conditional therapeutic recommendation. Ambiguous or non-committal statements were not permitted. All prompts were administered in German. The exact prompt is provided in the Supplementary Material (File S1). Each patient case was evaluated once per model, and no repeated testing of the same case was performed. All cases were entered sequentially within the same chat session for each respective model. Two LLMs were investigated: ChatGPT GPT-5.4 (Release date: 5 March 2026, OpenAI, San Francisco, CA, USA) and Claude Sonnet 4.6 (Release date: 17 February 2026, Antrophic, San Francisco, CA, USA). Acknowledging previous studies highlighting the importance of access to structured external clinical information for the quality of LLM-based medical recommendations, the latest versions of the respective LLM were used [18]. All LLMs were used in default mode, e.g., features such as “reasoning” were not employed. As each case was analyzed only once without repeated runs, formal assessment of intra-model output variability was not performed. To minimize temporal bias, all LLM prompts were explicitly framed to generate recommendations based solely on the clinical information available at the time of the original MTB presentation. No follow-up data or later clinical developments were provided to the models. LLM-generated recommendations were reviewed by study authors for categorization but not subjected to an independent qualitative assessment beyond comparison with the documented MTB recommendations.

### 2.2. Data Set Acquisition

**Figure 1:**
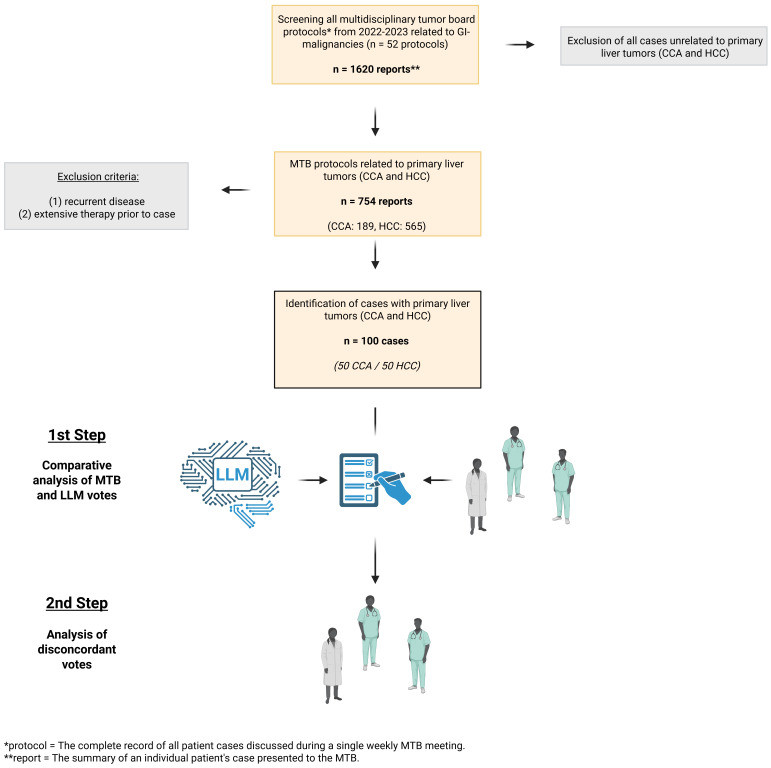
Schematic depiction of data set acquisition and the study design/workflow.

Seeking to explore the applicability of LLMs for oncologic decision making, a data set of MTB protocols concerning patients with newly diagnosed primary liver tumors who were at the beginning of their oncologic treatment was defined as the basis for the upcoming comparative analysis. Initially, MTB protocols discussed within the MTB at the University Hospital RWTH Aachen between 2022 and 2023 were screened for protocols pertaining to primary liver tumors. From 1456 MTB protocols, 754 discussed such tumor entities. Next, cases which met the following exclusion criteria were excluded: (1) recurrent disease, (2) extensive therapy in terms of multimodal treatment prior to case presentation at the MTB were excluded. Finally, 100 protocols of selected cases of primary liver tumors were identified. The extent of the clinical information available in the individual case records was not considered as inclusion or exclusion criteria in order to mimic real-world clinical practice. CCA and HCC cases accounted for equal amounts of 50 cases per tumor entity. The workflow used for case identification is demonstrated in Figure 1.

### 2.3. Statistical Analysis

Statistical analysis was performed to evaluate the agreement and comparative performance between MTB and the LLM system recommendations. Concordance between MTB and LLM decisions was evaluated using Cohen’s kappa (Κ) coefficient to quantify agreement beyond chance. Kappa values were interpreted according to Landis and Koch criteria: <0.00 poor, 0.00–0.20 slight, 0.21–0.40 fair, 0.41–0.60 moderate, 0.61–0.80 substantial, 0.81–1.00 almost perfect agreement [19]. Correlation analysis between MTB and LLM voting patterns was performed using Spearman’s rank correlation coefficient. Spearman’s rank correlation coefficient was selected to quantify the ordinal association between MTB and LLM treatment recommendations, as treatment categories represent ordered categorical outcomes rather than continuous variables. In addition, 95% confidence intervals were calculated using Fisher’s r-to-z transformation for all reported agreement statistics to facilitate interpretation of estimate precision. The strength of correlation was interpreted as follows: 0.00–0.19 very weak, 0.20–0.39 weak, 0.40–0.59 moderate, 0.60–0.79 strong, and 0.80–1.00 very strong. All statistical tests were two-tailed. A *p*-value < 0.05 was considered statistically significant, and a *p*-value < 0.001 was considered highly significant. All statistical analyses were performed using IBM SPSS Statistics, version 29.0.

## 3. Results

### 3.1. Cohort Characteristics

A total of 100 patients diagnosed with either CCA (*n* = 50) or HCC (*n* = 50) discussed in MTB meetings between 2022 and 2023 were included in the analysis. Descriptive analysis of the CCA cohort characteristics is presented in Table 1, while patient characteristics of HCC patients are summarized in Table 2. The CCA cohort was composed of 30 male patients (60%) and 20 female patients (40%) with a mean age at diagnosis of 63.9 ± 10.0 years, whereas the HCC cohort comprised 36 male patients (72%) and 14 female patients (28%), with a mean age of 68.5 ± 8.5 years. Most patients presented with a good performance status in both cohorts. Regarding CCA patients, an ECOG performance status of 0 was observed in 30 patients (60%), while 11 patients (22%) had an ECOG status of 1. Five patients (10%) had an ECOG status of 2, and no patients had an ECOG status of 3. ECOG data were unavailable for 4 patients (8%). In the HCC cohort, an ECOG score of 0 was documented for 20 patients (40%) and ECOG 1 for 15 patients (30%). Seven patients (14%) had an ECOG performance status of 2, while one patient (2%) had an ECOG score of 3. Two patients (4%) were classified as ECOG 4, and ECOG data were unavailable for five patients (10%).

In terms of CCA subtypes, pCCA, including Klatskin tumors, was the most common subtype, accounting for 24 cases (48%). ICCA was diagnosed in 18 patients (36%). DCCA and gallbladder carcinoma were each present in 3 patients (6%). In 2 cases (4%), the tumor subtype was not specified. Histological confirmation of the CCA tumor diagnosis was available in 31 patients (62%), whereas in 19 patients (38%), the diagnosis was established based on clinical and radiological findings without histopathological confirmation.

Regarding tumor stage classification, the distribution of patients diagnosed with HCC according to the Barcelona Clinic Liver Cancer (BCLC) staging system is summarized in Table 2. No patients were classified as BCLC stage 0. The majority of cases were assigned to early or intermediate disease stages, with 19 patients (38%) categorized as BCLC stage A and 15 patients (30%) as BCLC stage B. Advanced disease in terms of BCLC stage C was present in 11 patients (22%), while 5 patients (10%) were classified as terminal BCLC stage D.

### 3.2. Comparative Analysis of MTB and LLM Recommendations

#### 3.2.1. Agreement Between MTB and LLM Recommendations for Primary Liver Malignancies

Within the CCA cohort, statistical comparative analysis demonstrated a substantial agreement between MTB and ChatGPT treatment recommendations with a concordance rate of 80%, confirmed by a statistically highly significant (*p* < 0.001) Cohen’s kappa coefficient of 0.688 (standard error 0.083), indicating substantial concordance beyond chance. In addition, a strong positive correlation between MTB and LLM vote was observed, with a Spearman’s rank correlation coefficient of 0.725 (95% CI 0.553–0.837; *p* < 0.001), suggesting a consistent alignment of therapeutic prioritization between both approaches (Table 3). Interestingly, agreement between MTB and Claude turned out significantly lower with a concordance rate of 56% with a Cohen’s kappa coefficient of 0.425 (standard error 0.079; *p* < 0.001) and a moderate positive correlation as indicated by a Spearman’s rank correlation coefficient of 0.417 (95% CI 0.149–0.628; *p* = 0.003) (Table 3).

For HCC, comparison of therapeutic recommendations between the MTB and the ChatGPT system demonstrated substantial, yet less pronounced agreement compared to CCA, amounting to an overall exact agreement of 66%. Statistical analysis revealed a Cohen’s kappa coefficient of 0.604 (standard error 0.076; *p* < 0.001), indicating moderate-to-substantial concordance beyond chance. In addition, a statistically significant positive correlation was observed, with a Spearman’s rank correlation of 0.484 (95% CI 0.230–0.677; *p* < 0.001), reflecting consistent therapeutic recommendations between both approaches (Table 4). In contrast, agreement between the MTB and Claude was markedly lower. Exact concordance between Claude and the MTB was observed in 19 cases (38%). Correspondingly, statistical analysis demonstrated only fair agreement with a Cohen’s kappa coefficient of 0.314 (standard error 0.069; *p* < 0.001). Correlation analysis revealed no significant association between MTB and Claude recommendations, as indicated by a Spearman’s rank correlation coefficient of 0.086 (95% CI −0.205–0.363; *p* = 0.551), suggesting limited alignment of therapeutic prioritization between Claude-generated recommendations and MTB decisions (Table 4).

#### 3.2.2. Agreement Between MTB and LLM Recommendations

The agreement between MTB- and LLM-derived recommendations for patients is shown in Table 5 (CCA, ChatGPT) and Table 6 (CCA, Claude), as well as Table 7 (HCC, ChatGPT) and Table 8 (HCC, Claude) for patients diagnosed with CCA and HCC, respectively. Detailed cross-tabulations of MTB and LLM decisions are shown in Supplementary Tables S1–S4.

Concerning the CCA cohort, exact concordance between MTB and ChatGPT recommendations was observed in 40 of 50 cases (80%). Aside from complete agreement for *best supportive care* and *liver transplantation*, each represented by a single case (100%), the highest agreement rates were observed for *palliative systemic treatments* (10/11, 90.9%) and *surgery* (24/28, 85.7%). Overall, the distribution of recommendations across therapeutic categories was comparable between MTB and ChatGPT, with surgery (MTB: *n* = 28; ChatGPT: *n* = 25) and palliative systemic therapy (MTB: *n* = 11; ChatGPT: *n* = 15) being the most frequent votes (Supplementary Table S1).

Discrepancies between MTB and ChatGPT primarily occurred in cases involving *neoadjuvant systemic treatments* (33% agreement) and *supplementary diagnostics *(66.7% agreement). In most cases where the MTB recommended *neoadjuvant systemic treatments*, ChatGPT most frequently assigned patients to *palliative systemic treatments* (Supplementary Table S1).

Overall agreement between MTB and Claude recommendations was considerably lower compared to ChatGPT, with a total of 28 of 50 (56%) matching decisions (Table 6). Strikingly, the single most frequent recommendation contributed by Claude was *supplementary diagnostics* prior to any definitive treatment decision (*n* = 22). This was particularly interesting as ChatGPT recommended *supplementary diagnostics* only five times within the same cohort (Table 5). Naturally, when considering MTB as a reference standard, complete agreement was observed for all cases in which MTB recommended *supplementary diagnostics* (*n* = 3 or 100%). Complete agreement was also achieved for *best supportive care* and *liver transplantation*, each represented by a single case (*n* = 1, 100%). Agreement for the remaining treatment categories was the highest for *palliative systemic treatment* (6/11, 90.9%) and *surgery* (15/28, 85.7%) (Supplementary Table S2). Again, recommendations regarding *neoadjuvant systemic treatment* showed the lowest agreement rate (33.3%).

Acknowledging the greater variety of eligible therapeutic options compared to CCA, numeric concordance measures were notably lower in the HCC group (Table 7). In general, the distribution of treatment recommendation across therapeutic categories was comparable between MTB and ChatGPT, with *surgery* (MTB: *n* = 10; ChatGPT: *n* = 9), *local therapy* (MTB: *n* = 6; ChatGPT: *n* = 17), *systemic treatment *(MTB: *n* = 10; ChatGPT: *n* = 9), and *transplant-related strategies* (*bridge to transplant* and *transplantation* combined: MTB: *n* = 13; ChatGPT: *n* = 9) representing the most frequent treatment allocations (Supplementary Table S3). Regarding agreement between MTB and ChatGPT recommendations concerning HCC patients, exact concordance, was observed in 33 of 50 cases (66%). Aside from complete agreement for *local ablative* or *interventional therapy *(6/6, 100%), and *best supportive case* (1/1, 100%), the highest agreement rates were observed for *systemic treatment* (8/10, 80%), followed by *bride to transplant* (5/7, 71.4%), *transplantation* (4/6, 66.7%) and *surgery* (6/10, 60%). The lowest agreement rates were observed for *radiotherapy *(1/3, 33.3%), *combined chemo + local therapy* (1/4, 25%), and *watch-and-wait* (1/3, 33.3%) strategies, with each single concordant case. Discrepancies between MTB and ChatGPT recommendations primarily occurred in intermediate-stage treatment categories, where ChatGPT tended to assign patients more frequently to *local therapy approaches*, whereas the MTB more often favored multimodal or individualized sequential treatment strategies, including liver transplantation as the ultimate treatment goal (Supplementary Table S3).

Agreement between MTB and Claude was substantially lower compared to ChatGPT, with concordant treatment recommendations overall in 19 of 50 cases (38%) (Table 8). Again, complete agreement was observed for the *best supportive case* (1/1, 100%). Highest agreement rates were documented for *bridge to transplant* (5/7, 71.4%) and *systemic therapies* (5/10, 50%), followed by surgery (4/10, 40%). In all other categories, very low agreement rates were registered.

Consistent with the trend observed in the CCA cohort, Claude most frequently recommended *supplementary diagnostics* (*n* = 17), whereas the MTB more commonly assigned patients to *surgery* (*n* = 10) or *systemic treatment* (*n* = 10) (Supplementary Table S4). Interestingly, in contrast to ChatGPT, Claude also recommended *bridge to transplantation* substantially more frequently, generating twice as many recommendations as the MTB (*n* = 14 vs. *n* = 7) and almost three times as many recommendations as ChatGPT (*n* = 14 vs. *n* = 5; Supplementary Table S3).

#### 3.2.3. Exploration of Discordant MTB and LLM Recommendations

In general, MTB and ChatGPT recommendations regarding CCA cases showed good concordance (Table 3). Yet, in a few cases, the ChatGPT vote differed from the MTB vote (Table 5 and Table 9). In total, 10 decisions did not match (Table 9). In four cases (*n* = 4), ChatGPT yielded towards more restrictive OP indications, stating various reasons: high-perioperative risk (*n* = 1), contraindication for surgery due to initial distant metastasis despite to-date tumor-regress under *systemic treatment* (*n* = 1), stating the demand for histological confirmation prior to a final decision or rather no resection upon histological confirmation (*n* = 1) or demand for other diagnostic measures indicated prior to surgical intervention (*n* = 1). Among the four patients who underwent surgery in accordance with the MTB vote at the time, two received curative R0-resection and experienced no significant postoperative complications (Clavien–Dindo ≥ 3a). As of this date, both patients remain alive. The remaining two patients proceeded to surgical exploration but were found to be inoperable intraoperatively and were, therefore, deemed ineligible for surgical therapy. One of these patients encountered a complicated postoperative course, with complications classified as Clavien–Dindo ≥ 3a due to pre-existing comorbidities. However, this patient was ultimately discharged in good condition with options for further treatment. Unfortunately, the two patients who had undergone surgical exploration but were not eligible for curative surgery have since passed away.

In contrast, in one case, ChatGPT clearly recommended *surgery* due to given resectability, while MTB decided on *neoadjuvant systemic treatment* (*n* = 1). Furthermore, in one case, ChatGPT did not state the demand for *histological confirmation,* while MTB’s decision insisted on a histological tumor diagnosis confirmation prior to a final vote (*n* = 1). Regarding *neoadjuvant or palliative systemic treatment,* ChatGPT-based decisions categorically excluded patients with distant metastasis from *neoadjuvant treatment*, directing them towards *palliative treatment*, whereas MTB presented more flexible therapeutic decisions in terms of re-evaluation of resectability upon *neoadjuvant systemic treatment* (*n* = 3). Interestingly, an inverse decision path for either entity was observed, where MTB decided upon *palliative systemic treatment,* while ChatGPT-based decisions favored *neoadjuvant systemic treatment* with re-evaluation of surgical therapy options within the course of the treatment (*n* = 1).

Table 10 demonstrates the distribution of recommendations provided by Claude in cases where MTB recommended alternative management strategies. Among these discordant cases, Claude most frequently suggested supplementary cases (19/22 cases), followed by *palliative systemic treatment *(*n* = 2) and *local therapies *(*n* = 1). Upon *palliative treatment* recommendations suggested by Claude, the corresponding MTB recommendations included *surgery* (*n* = 1) and *neoadjuvant systemic treatment *(*n* = 1). Overall, the majority of discordant cases were characterized by Claude favoring additional diagnostic work-up rather than immediate intervention.

MTB and ChatGPT recommendations regarding HCC cases demonstrated moderate concordance. Nevertheless, a relevant number of discordant decisions were observed, with a total of 17 mismatching recommendations between MTB and ChatGPT (Table 11).

Among these, discrepancies were most frequently observed in cases where the MTB recommended *local therapies* (*n* = 11). In this subgroup, ChatGPT suggested a broad range of alternative strategies, including *surgical resection* (*n* = 3), *systemic treatment* (*n* = 1), *radiotherapy* (*n* = 2), combined *chemo-local approaches* (*n* = 2), *bridging to transplantation* (*n* = 2), and *watchful waiting* (*n* = 1), reflecting a diversification of treatment options in intermediate-stage disease.

In cases where the MTB recommended *surgical resection* (*n* = 3), ChatGPT did not propose surgery in any instance but instead favored *bridging strategies* (*n* = 2) or *combined treatment approaches* (*n* = 1), suggesting a more conservative or stepwise approach. Conversely, in a limited number of cases, ChatGPT recommended surgical intervention while the MTB opted for non-surgical strategies, including *local therapies* (*n* = 3) or *systemic treatment* (*n* = 1).

Further discordance was observed in cases involving *systemic treatment* or *BSC*. In one case, the MTB recommended *systemic treatment*, whereas ChatGPT suggested *surgical treatment* (*n* = 1). In two cases with MTB recommendations for *BSC*, ChatGPT proposed either *systemic treatment* or a *watch-and-wait strategy* (*n* = 2), indicating differing assessments of treatment eligibility.

Analysis of discordant recommendations generated by Claude revealed a distinct pattern compared to ChatGPT (Table 12). A substantial proportion of discordant cases were characterized by Claude recommending *supplementary diagnostics* (*n* = 17), reflecting a cautious approach with preference for deferral of definitive treatment decisions.

In cases where the MTB recommended *bridging to transplantation* (*n* = 9), Claude suggested a wide range of alternative strategies, including *surgery* (*n* = 1), *local therapies* (*n* = 2), *LTX* (*n* = 3), *combined approaches* (*n* = 2), and *watchful waiting* (*n* = 1). Similarly, in cases with MTB recommendations for *surgery* (*n* = 3), Claude favored *bridging strategies* (*n* = 2) or *radiotherapy* (*n* = 1).

## 4. Discussion

The implementation of LLMs in clinical oncology has introduced new opportunities to support decision-making processes [20]. This may be of particular use within the setting of MTBs [21,22]. As MTBs represent the reference standard in oncologic treatment planning, any digital or LLM-based adjunct must be critically assessed for clinical accuracy, applicability, safety and alignment with current evidence-based guidelines. In this study, we retrospectively compared treatment recommendations provided by an established MTB to those generated by LLMs (ChatGPT and Claude) for newly diagnosed cases of primary liver tumors, including CCA and HCC. Importantly, MTB recommendations were used as a real-world clinical comparator rather than an absolute gold standard, as treatment decisions may vary between institutions and individual expert panels.

In general, several trends could be observed. For one, overall concordance rates among both LLMs were notably higher for CCA than for HCC. This difference may be attributed to a broader range of therapeutic options for HCC compared to CCA [23]. Second, ChatGPT produced markedly higher concordance rates than Claude in both cohorts.

Although this study was not designed to investigate the underlying mechanisms responsible for the observed differences, one possible explanation may relate to differences in model design, training and alignment strategies, and safety frameworks. Although both systems are general-purpose LLMs, they are developed by different organizations with distinct training paradigms and alignment strategies. Claude, developed by Anthropic, is trained using the concept of *Constitutional AI*, an alignment approach that embeds a predefined set of ethical principles into the training process to ensure that output remains helpful, harmless and adherent to strong safety constraints [24]. This framework aims to minimize potentially harmful or ethically questionable responses and often leads to more cautious or conservative outputs, particularly in sensitive domains such as healthcare. This cautious approach to medical decision-making was particularly evident as Claude demanded additional diagnostics in order to affirm the suspected diagnosis prior to a definitive recommendation much more frequently than ChatGPT (CCA cohort: *n* = 22 vs. *n* = 5; Table 5 and Table 6).

In contrast, ChatGPT is primarily trained using *reinforcement learning from human feedback* (*RLHF*), where human annotators rank model responses to optimize helpfulness and task completion [25,26]. While this approach also incorporates safety guardrails, it is generally designed to maximize usefulness and responsiveness to user prompts, which may result in more decisive or directive outputs when applied to clinical decision scenarios.

These differences in alignment philosophy may translate into distinct behavioral tendencies when models are applied to medical decision-making tasks. Safety-oriented models such as Claude have been reported to show higher tendency toward cautious or restrained responses, including avoidance of definitive medical advice or preference for safer, guideline-conservative options. Such “safety-pessimism” or over-refusal behavior has also been described in recent evaluations of healthcare-oriented LLM benchmarks, where strongly safety-aligned models may prioritize risk minimization over decisiveness [27,28].

In general, agreement between MTB and LLM recommendations for patients diagnosed with CCA was substantial, particularly for ChatGPT. Although exceeding some previously described [21], the agreement between MTBs and ChatGPT recommendations remained within the range commonly reported for medical-decision making [29,30]. However, subtle differences in decision patterns between MTB and ChatGPT were observed for specific subgroups.

Overall, MTB decisions within the CCA cohort demonstrated a broader willingness to a more liberal indication for surgery compared to the more guideline-constrained recommendations provided by ChatGPT. The exact reasons for deviations from national and international guidelines in MTB decisions that diverged from ChatGPT’s recommendations remain unclear. However, several contributing factors are highly likely. First, eligibility for R0 resection may be determined through interdisciplinary evaluation by experienced physicians, especially surgeons and radiologists, even in cases that formally meet the criteria for systemic disease—such as imaging-based suspicion of distant lymph node metastasis—thus maintaining a curative intent despite guideline-defined advanced stage. Second, the higher frequency of *neoadjuvant approaches* in MTB decisions, as compared to ChatGPT’s tendency to assign patients directly to *palliative systemic treatment*, may reflect a human inclination to preserve options for future reassessment rather than prematurely define a case as palliative. Third, the actual clinical condition of the patient, which may not be fully captured by ECOG performance status alone, can influence treatment decisions. Collectively, these factors suggest that MTB decisions reflect an individualized treatment philosophy that extends beyond rigid guideline adherence.

Follow-up data of patients diagnosed with CCA who underwent surgical treatment in accordance with MTB recommendations, and despite the respective ChatGPT-vote, revealed interesting insights. Half of these patients experienced a clear benefit from the MTB decision, in terms of curative R0-resection without significant postoperative complications (Clavien–Dindo ≥ 3a). Hypothetically, if exclusively ChatGPT-based decision-making had been applied, these patients would likely have received *systemic treatment*, corresponding to a palliative rather than curative treatment approach. The reported clinical outcomes should be regarded as descriptive observations rather than superior clinical utility. Formal survival analyses and outcome comparisons between concordant and discordant cases were beyond the scope of the present study.

Within the HCC cohort, patients for whom the MTB recommended surgical resection, despite a differing ChatGPT vote, revealed similar patterns, along with additional noteworthy findings. Among the four patients for whom the MTB initially recommended surgical resection, two ultimately underwent surgery with curative R0 resection with an uneventful postoperative course. In the remaining two cases, the initially planned surgical strategy was not pursued during the subsequent clinical course. Instead, one patient received radiotherapy, while the other underwent transarterial chemoembolization (TACE), the latter corresponding to the treatment strategy previously suggested by ChatGPT. These observations illustrate that MTB decisions enabled curative treatment in a subset of patients, while the dynamic nature of oncologic decision-making and the availability of additional clinical information during follow-up occasionally resulted in therapeutic adjustments that coincided with alternative strategies previously proposed by ChatGPT.

Importantly, the present study is based on individualized real-world patient cases derived from routine clinical care at a tertiary referral center, including comprehensive longitudinal follow-up and detailed clinical annotation. Unlike analyses relying on large registry data sets or synthetic benchmark cohorts, our approach reflects authentic clinical complexity, including comorbidities, treatment tolerability, institutional workflows, and evolving therapeutic strategies. This real-world context strengthens the clinical relevance of our findings and provides a robust framework for evaluating LLM-assisted decision-support under practical conditions.

This aspect is particularly relevant in the management of HCC, where therapeutic standards are rapidly evolving and may vary across geographic regions, healthcare systems, and guideline bodies. In this context, ChatGPT may reflect patterns learned from a broad body of guideline-based and clinical literature, which may support structured treatment recommendations.

In contrast, the second evaluated LLM, Claude, demonstrated a noticeably different recommendation pattern. Compared with ChatGPT, Claude generated more cautious responses and more frequently proposed additional diagnostic evaluation rather than committing to a definitive treatment recommendation. This was reflected in a substantially higher number of cases requiring further diagnostic work-up prior to therapeutic allocation. While this approach may reflect a conservative reasoning strategy aimed at reducing the risk of inappropriate treatment selection in the presence of uncertainty, it also resulted in a markedly lower concordance rate with MTB decisions.

Additionally, Claude tended to present a broader range of potential therapeutic options rather than prioritizing a single strategy, which may further reduce direct agreement with MTB recommendations. In contrast, ChatGPT consistently provided clear and decisive therapeutic guidance. The lack of such decisiveness in Claude’s responses may partially explain the lower observed concordance with the MTB decision.

From a workflow perspective, integrating LLM-generated treatment suggestions as a preparatory component of MTB discussions could support structured clinical reasoning and enhance consistency in therapeutic evaluations. Rather than replacing expert judgment, such systems could function as adjunctive tools, facilitating more comprehensive discussions and serving as a second-opinion framework. Future prospective studies should evaluate whether real-time integration of LLM-assisted recommendations into MTB workflows can improve decision efficiency, reduce time to treatment, and enhance guideline adherence without compromising patient safety. This concept is consistent with the vision proposed by Nault et al., who described the emergence of a “next-generation” liver tumor board integrating artificial intelligence and precision oncology approaches into MTB decision-making processes [31]. Taken together, these findings provide important insights into the role of LLMs in multidisciplinary oncologic decision-making. The observed level of concordance between MTB and ChatGPT indicates that LLM-based systems are capable of generating clinically meaningful, guideline-consistent therapeutic suggestions in complex real-world cases. Importantly, as demonstrated by Yang et al., in their investigation of concordance between LLM-generated and physician-derived treatment recommendations, decisions for patients with HCC, concordance did not necessarily translate into improved clinical outcome [32]. Moreover, the observation that ChatGPT recommendations occasionally anticipated later MTB decisions, suggests a potential role in early therapeutic orientation and structured case preparation. Finally, differences between LLMs highlight that AI systems exhibit distinct decision-making behaviors, ranging from more decisive guideline-driven recommendation patterns to more cautious, diagnostic-oriented strategies. Understanding these behavioral differences will be important for the future integration of AI-based systems into clinical workflows. Importantly, despite the promising agreement observed in the present study, LLM-generated recommendations remain susceptible to hallucinations, incomplete reasoning, and incorrect treatment suggestions. Consequently, and on behalf of patient safety aspects, these systems should be regarded exclusively as decision-support tools requiring expert oversight rather than autonomous clinical decision-makers. Still, the controlled implementation of LLMs in clinical decision-making may augment multidisciplinary decision-making through improved access to aggregated medical knowledge and support in navigating increasingly complex therapeutic landscapes.

## 5. Limitations

Several limitations should be considered when interpreting these findings. First, the retrospective single-center design limits generalizability, as institutional practices, case mix, and local treatment algorithms may differ across centers. Within this context, despite predefined inclusion criteria, a potential risk of selection bias cannot be excluded. Second, acknowledging the relatively small cohort size, these findings should be interpreted as exploratory and hypothesis-generating rather than definitive evidence. Future studies incorporating larger patient cohorts and external validations are warranted to further assess the generalizability and robustness of these findings. Third, LLM-based recommendations were generated using structured case summaries rather than full access to primary imaging, pathology slides, laboratory trends, and longitudinal clinical context, all of which are integral to real-world MTB decision-making. Consequently, concordance rates and apparent anticipatory recommendations may partly reflect simplified data representations rather than true clinical equivalence. Fourth, a potential temporal bias must be acknowledged, as the MTB cases originated from 2022–2023, whereas the evaluated LLMs represent later model versions that may incorporate more recent guidelines, clinical trial data, or updated treatment standards. This may have conferred a structural advantage to the models in selected cases. Future prospective studies should therefore aim for contemporaneous comparisons or guideline-date restricted prompting strategies. Fifth, LLM outputs depend critically on prompt structure, information completeness, and model versioning, all of which may affect reproducibility and consistency. Moreover, recommendations generated by LLMs are inherently stochastic and may vary across repeated evaluations of the same clinical case. Since each patient was assessed only once in the present study, recommendation stability and reproducibility could not be evaluated. Future studies should therefore investigate inter-run variability and assess the robustness of LLM-derived treatment recommendations across multiple independent evaluations. In addition, all cases were entered sequentially within a continuous chat session to ensure a standardized evaluation workflow while also mimicking routine clinical use, in which LLMs may be employed continuously across multiple patient cases rather than in isolated sessions. Although identical prompting was maintained throughout the study, contextual information from preceding cases may theoretically have influenced subsequent recommendations. Future investigations should compare sequential and isolated case evaluation strategies to quantify potential context contamination effects. Finally, current LLMs lack true clinical reasoning, contextual awareness, and accountability, emphasizing that their role should remain strictly supportive. Prospective, multicenter validation studies with standardized data input, blinded comparative evaluation, and assessment of clinical outcomes, and complementary analyses of guideline concordance will be essential to define the true utility, safety, and ethical implications of integrating LLMs into oncologic decision-support systems.

## 6. Conclusions

This retrospective single-center study demonstrates substantial concordance between MTB treatment recommendations and those generated by LLMs for patients with primary liver malignancies. Notably, different LLMs showed marked differences in both concordance rates and response characteristics, indicating that the choice of model itself represents an important factor influencing AI-assisted decision-support performance. These findings underscore the complementary nature of LLM-assisted systems, which may enhance efficiency, consistency, and guideline adherence without replacing expert judgement.

Prospective multicenter studies are warranted to validate these results and to define the impact of real-time LLM integration on clinical workflows, decision quality, and patient outcomes.

## Figures and Tables

**Table 1:** CCA cohort characteristics.

Characteristic	Distribution (*n* (±SD))	Distribution (%)
Gender		
Male	30	60
Female	20	40
Age		
Age (years)	63.9 ± 10.0	-
ECOG		
0	30	60
1	11	22
2	5	10
3	0	0
N/A	4	8
CCA-Subtype		
iCCA	18	36
pCCA or Klatskin tumor	24	48
dCCA	3	6
Gallbladder-Ca	3	6
Not specified	2	4
Histological confirmation of tumor diagnosis		
Yes	31	62
No	19	38

**Table 2:** HCC cohort characteristics.

Characteristic	Distribution (*n* (±SD))	Distribution (%)
Gender		
Male	36	72
Female	14	28
Age		
Age (years)	68.5 ± 8.5	-
ECOG		
0	20	40
1	15	30
2	7	14
3	1	2
4	2	4
N/A	5	10
BCLC		
0	0	0
A	19	38
B	15	30
C	11	22
D	5	10

**Table 3:** Statistical analysis of concordance and correlation between MTB and LLM recommendations for CCA.

LLM	Statistical Measure	Value	Standard Error	Significance Level	Confidence Interval (CI)
ChatGPT	Exact Agreement (between LLM and MTB)	*n* = 40 (80%)	-	-	-
	Cohen’s Kappa (concordance)	0.688	0.083	<0.001	-
	Spearman (correlation)	0.725	-	<0.001	0.553–0.837
Claude	Exact Agreement (between LLM and MTB)	*n* = 28 (56%)	-	-	-
	Cohen’s Kappa (concordance)	0.425	0.079	<0.001	-
	Spearman (correlation)	0.417	-	0.003	0.149–0.628

**Table 4:** Statistical analysis of concordance and correlation between MTB and LLM recommendations for HCC.

LLM	Statistical Measure	Value	Standard Error	Significance Level	Confidence Interval
ChatGPT	Exact Agreement (between LLM and MTB)	*n* = 33 (66%)	-	-	-
	Cohen’s Kappa (concordance)	0.604	0.076	<0.001	-
	Spearman (correlation)	0.484	-	<0.001	0.230–0.677
Claude	Exact Agreement (between LLM and MTB)	*n* = 19 (38%)	-	-	-
	Cohen’s Kappa (concordance)	0.314	0.069	<0.001	-
	Spearman (correlation)	0.086	-	0.551	−0.205–0.363

**Table 5:** Agreement between MTB and ChatGPT recommendations for CCA.

MTB Recommendation	Concordant	Discordant	Agreement (%)
Surgery	24	4	85.7
Neoadjuvant systemic treatment	2	4	33.3
Palliative systemic treatment	10	1	90.9
Supplementary diagnostics	2	1	66.7
Best supportive care	1	0	100.0
Liver transplantation	1	0	100.0
Total	40	10	80.0

**Table 6:** Agreement between MTB and Claude recommendations for CCA.

MTB Recommendation	Concordant	Discordant	Agreement (%)
Surgery	15	13	53.6
Neoadjuvant systemic treatment	2	4	33.3
Palliative systemic treatment	6	5	54.5
Supplementary diagnostics	3	0	100.0
Best supportive care	1	0	100.0
Liver transplantation	1	0	100.0
Total	28	22	56.0

**Table 7:** Agreement between MTB and ChatGPT recommendations for HCC.

MTB Recommendation	Concordant	Discordant	Agreement (%)
Surgery	6	4	60.0
Local therapy *	6	0	100.0
Systemic treatment	8	2	80.0
Bridge to transplantation	5	2	71.4
Transplantation	4	2	66.7
Best supportive care	1	0	100.0
Radiotherapy	1	2	33.3
Combined chemo + local therapy	1	3	25.0
Watch and wait	1	2	33.3
Total	33	17	66.0

* Including Ablation (MWA, RFA, IRE) and TACE.

**Table 8:** Agreement between MTB and Claude recommendations for HCC.

MTB Recommendation	Concordant	Discordant	Concordance (%)
Surgery	4	6	40.0
Local therapy *	1	5	16.7
Systemic treatment	5	5	50.0
Bridge to transplant	5	2	71.4
Transplant	1	5	16.7
Best supportive care	1	0	100.0
Radiotherapy	1	2	33.3
Combined chemo + local therapy	0	4	0.0
Watch and wait	1	2	33.3
Total	19	31	38.0

* Including Ablation (MWA, RFA, IRE) and TACE.

**Table 9:** Cross-table of discordant MTB and ChatGPT vote for CCA.

MTB/ChatGPT	Surgery	Neoadjuvant Systemic Treatment	Palliative Systemic Treatment	Supplementary Diagnostics	Total
Surgery	0	0	2	2	4
Neoadjuvant systemic treatment	0	0	3	1	4
Palliative systemic treatment	0	1	0	0	1
Supplementary diagnostics	1	0	0	0	1
Total	1	1	5	3	10

**Table 10:** Cross-table of discordant MTB and Claude votes for CCA.

MTB/Claude	Surgery	Neoadjuvant Systemic Treatment	Palliative Systemic Treatment	Total
Palliative Systemic Treatment	1	1	0	2
Local Therapy	1	0	0	1
Supplementary Diagnostics	11	3	5	19
Total	13	4	5	22

**Table 11:** Cross-table of discordant MTB and ChatGPT votes for HCC.

MTB/ChatGPT	Surgery	Systemic Treatment	Bridge to Tx	Tx	Radiotherapy	Combined Chemo + Local Therapy	Watch and Wait	Total
Surgery	0	0	2	0	0	1	0	3
Local therapy	3	1	0	2	2	2	1	11
Systemic treatment	1	0	0	0	0	0	0	1
BSC	0	1	0	0	0	0	1	2
Total	4	2	2	2	2	3	2	17

Tx = Transplantation.

**Table 12:** Cross-table of discordant MTB and Claude votes for HCC.

MTB/Claude	Surgery	Local Therapy	Systemic Treatment	Bridge to Tx	Tx	Radiotherapy	Combined Chemo + Local Therapy	Watch and Wait	Total
Surgery	0	0	0	2	0	1	0	0	3
Bridge to Tx	1	2	0	0	3	0	2	1	9
Radiotherapy	0	0	1	0	0	0	0	0	1
BSC	0	0	1	0	0	0	0	0	1
Supplementary Diagnostics	5	3	3	0	2	1	2	1	17
Total	6	5	5	2	5	2	4	2	31

Tx = Transplantation.

## Data Availability

All data generated or analyzed during this study are included in this article.

## Supplementary Materials

The following supporting information can be downloaded at: https://www.mdpi.com/article/10.3390/cancers18132175/s1, File S1: Complete prompt template; Table S1. Cross-table of MTB and ChatGPT recommendations for CCA; Table S2. Cross-table of MTB and Claude recommendations for CCA; Table S3. Cross-table of MTB and ChatGPT recommendations for HCC; Table S4. Cross-table of MTB and Claude recommendations for HCC.
