# Supplementary material for: Large Language Models for Multidisciplinary Tumor Board Decision-Making in Primary Liver Tumors: A Retrospective Single-Center Study
Source: Cancers (Basel). 2026 Jul 7;18(13):2175. doi: 10.3390/cancers18132175 (PMC13359904; doi:10.3390/cancers18132175)
Supplement: Supplementary file 1 [file cancers-18-02175-s001.zip › cancers-4399573-supplementary.pdf]

Supplementary Material

File S1. Complete Prompt template.

The prompt used for both GPT-5.4 and Claude Sonnet 4.6 was administered in German.

The original prompt used was:

„In diesem Chat werden wie bei einer interdisziplinären Tumorkonferenz onkologische Fallvignetten präsentiert. Deine Aufgabe ist es, als Ersatz für eine interdisziplinäre Tumorkonferenz, die Fälle zu analysieren und auf Basis aktueller nationaler und internationaler Leit- und Richtlinien Therapieempfehlungen für jeden speziellen Fall eine individuelle Therapieempfehlung auszusprechen. Ziel ist eine klare und präzise Formulierung einer klaren Therapieempfehlung bzw. eines onkologischen Therapiekonzeptes, vage oder abwägende Aussagen sind nicht akzeptabel.

Akzeptable Empfehlungen umfassen:

1) klare Therapieempfehlungen oder Entscheidungen für ein klares Therapiekonzept
2) "falls, dann"-Entscheidungen, sprich "falls Befund A positiv/negativ, dann wird die Therapie XY eingeleitet" (oder ähnlich)
3) Anforderung weiterer Diagnostik, sofern dringend erforderlich und unabdingbar für eine Therapieempfehlung
4) Eine Kombination aus 3) und 1) oder 3) und 2)

Hast du die Aufgabe verstanden?

English Translation:

In this chat, oncological case vignettes will be presented in a format similar to that of a multidisciplinary tumor board. Your task is to act as a substitute for a multidisciplinary tumor board by analyzing each case and, based on current national and international clinical guidelines, providing an individualized treatment recommendation for each specific patient. The goal is to formulate a clear, precise, and unambiguous treatment recommendation or oncological management plan. Vague or equivocal statements are not acceptable.

Acceptable recommendations include:

1. Clear treatment recommendations or decisions outlining a definitive therapeutic strategy.
2. Conditional ("if–then") recommendations, e.g., "If finding A is positive/negative, then treatment X should be initiated" (or equivalent).
3. Recommendation of additional diagnostic investigations, but only if they are urgently required and indispensable for making a treatment recommendation.
4. A combination of (3) and (1), or (3) and (2).

Have you understood the task?"

Table S1. Cross-table of MTB and ChatGPT recommendations for CCA.

MTB / ChatGPT	Surgery	Neoadjuvant systemic therapy	Palliative systemic treatment	Supplementary diagnostics	BSC	LTX	To tal
**Surgery**	24	0	2	2	0	0	28
**Neoadjuvant systemic therapy**	0	2	3	1	0	0	6
**Palliative systemic treatment**	0	1	10	0	0	0	11
**Supplementary diagnostics**	1	0	0	2	0	0	3
**BSC**	0	0	0	0	1	0	1
**LTX**	0	0	0	0	0	1	1
**Total**	25	3	15	5	1	1	50

MTB = row; ChatGPT = column.

Table S2. Cross-table of MTB and Claude recommendations for CCA

MTB / Claude	Surgery	Neoadjuvan t systemic therapy	Palliative systemic treatment	Local therapies	Supplementar y diagnostics	BSC	LTX	Tota l
**Surgery**	15	0	1	1	11	0	0	28
**Neoadjuvant systemic therapy**	0	2	1	0	3	0	0	6
**Palliative systemic treatment**	0	0	6	0	5	0	0	11
**Supplementar y diagnostics**	0	0	0	0	3	0	0	3
**Best supportive care**	0	0	0	0	0	1	0	1
**Liver transplantatio n**	0	0	0	0	0	0	1	1
**Total**	15	2	8	1	22	1	1	50

MTB = row; Claude = column.

Table S3. Cross-table of MTB and ChatGPT recommendations for HCC.

MTB / ChatGPT	Sur-gery	Local ther-apy*	Sys-temic treat-ment	Bridge to Tx	Tx	BSC	Radio-therapy	Com-bined chemo + local therapy	Watc h and wait	To-tal
Surgery	6	3	1	0	0	0	0	0	0	10
Local therapy*	0	6	0	0	0	0	0	0	0	6
Systemic treatment	0	1	8	0	0	1	0	0	0	10
Bridge to Tx	2	0	0	5	0	0	0	0	0	7
Tx	0	2	0	0	4	0	0	0	0	6
BSC	0	0	0	0	0	1	0	0	0	1
Radio-therapy	0	2	0	0	0	0	1	0	0	3
Com-bined chemo + local therapy	1	2	0	0	0	0	0	1	0	4
Watch and wait	0	1	0	0	0	1	0	0	1	3
Total	9	17	9	5	4	3	1	1	1	50

MTB = row; ChatGPT = column; * Including Ablation (MWA, RFA, IRE) and TACE; Tx = Transplant.

Table S4. Cross-table of MTB and Claude recommendations for HCC.

MTB / Claude	Sur-gery	Local ther-apy*	Sys-temic treat-ment	Bridge to Tx	Tx	Radio-therapy	Com-bined chemo + local therapy	BSC	Watch and wait	Supple-mentary diagnos-tics	Total
Surgery	4	0	0	1	0	0	0	0	0	5	10
Local therapy*	0	1	0	2	0	0	0	0	0	3	6
Systemic treatment	0	0	5	0	0	1	0	1	0	3	10
Bridge to Tx	2	0	0	5	0	0	0	0	0	0	7
Tx	0	0	0	3	1	0	0	0	0	2	6
Radio-therapy	1	0	0	0	0	1	0	0	0	1	3
Combined chemo + local ther-apy	0	0	0	2	0	0	0	0	0	2	4
Best sup-portive care	0	0	0	0	0	0	0	1	0	0	1
Watch and wait	0	0	0	1	0	0	0	0	1	1	3
Total	7	1	5	14	1	2	0	2	1	17	50

MTB = row; Claude = column; * Including Ablation (MWA, RFA, IRE) and TACE; Tx = Transplant.
